# No neuroprotective effect of therapeutic hypothermia following lipopolysaccharide-sensitized hypoxia-ischemia: a newborn piglet study

**DOI:** 10.3389/fped.2023.1268237

**Published:** 2023-11-28

**Authors:** Mads Andersen, Hannah Brogård Andersen, Ted Carl Kejlberg Andelius, Lærke Hjøllund Hansen, Regitze Pinnerup, Mette Bjerre, Steffen Ringgaard, Leslie Schwendimann, Pierre Gressens, Kasper Jacobsen Kyng, Tine Brink Henriksen

**Affiliations:** ^1^ Department of Paediatrics and Adolescent Medicine, Aarhus University Hospital, Aarhus, Denmark; ^2^ Department of Clinical Medicine, Aarhus University, Aarhus, Denmark; ^3^ Medical Research Laboratory, Aarhus University, Aarhus, Denmark; ^4^ MR Research Centre, Aarhus University Hospital, Aarhus, Denmark; ^5^ Université Paris Cité, Inserm, NeuroDiderot, F-75019, Paris, France

**Keywords:** hypoxic-ischemic encephalopathy, lipopolysaccharide, therapeutic hypothermia, neuroprotection, animal model

## Abstract

**Introduction:**

Therapeutic hypothermia is the only proven neuroprotective treatment for hypoxic-ischemic encephalopathy. However, studies have questioned whether therapeutic hypothermia may benefit newborns subjected to infection or inflammation before a hypoxic-ischemic insult. We aimed to compare newborn piglets with lipopolysaccharide-sensitized hypoxia-ischemia treated with and without therapeutic hypothermia with regards to measures of neuroprotection.

**Methods:**

A total of 32 male and female piglets were included in this randomized experimental study. Lipopolysaccharides from *Escherichia coli* were infused intravenously before initiation of a standardized global hypoxic-ischemic insult. The piglets were then randomized to either normothermia or therapeutic hypothermia. After 14 h, the piglets were evaluated. Our primary outcome was brain lactate/N-acetylaspartate ratio assessed by magnetic resonance spectroscopy. Secondary outcomes included measures of magnetic resonance imaging, amplitude-integrated electroencephalography, immunohistochemistry, and concentration of blood cells and cytokines.

**Results:**

Piglets treated with and without therapeutic hypothermia were subjected to comparable global hypoxic-ischemic insults. We found no difference between the two groups with regards to measures of magnetic resonance spectroscopy and imaging, amplitude-integrated electroencephalography, immunohistochemistry, and concentration of blood cells and cytokines.

**Conclusion:**

We found no indication of neuroprotection by therapeutic hypothermia in newborn piglets following lipopolysaccharide-sensitized hypoxia-ischemia. However, interpretation of the results is limited by the short observation period. Further studies are required to determine the potential clinical implications of these findings.

## 1. Introduction

Neonatal encephalopathy due to intrapartum-related events is a severe clinical condition that affects over one million newborns each year (1). Hypoxia-ischemia (HI) is a common cause leading to what is termed hypoxic-ischemic encephalopathy (2). Therapeutic hypothermia (TH) is the only current neuroprotective treatment for hypoxic-ischemic encephalopathy, proven to be beneficial in several clinical randomized controlled trials (3). However, around 40%–50% of newborns treated with TH still die or suffer neurodevelopmental impairment (4, 5). Studies from low-income countries even suggest that TH may have adverse effects in these settings (6–8). Therefore, studies are needed to investigate why this treatment not always is associated with neuroprotection.

It has been proposed that perinatal infection and inflammation may act together with HI to create significant injury in the developing brain (9). Animal studies have found that sensitization before HI by bacterial endotoxins such as lipopolysaccharides (LPS) from *Escherichia coli* severely exacerbates newborn brain injuries (10–15). A previous piglet study found that LPS sensitization compared with HI alone affected brain expression of inflammatory markers and increased whole-brain cell death and mortality—with similar findings observed in several rat studies (10–15). This has been termed *the multiple hit hypothesis*, which postulates that an insult during pregnancy or labor may sensitize the fetal or neonatal brain for secondary insults to have larger clinical impact (9). In addition to the possible exacerbation of brain injury, animal studies have found TH with limited neuroprotective effect following LPS-sensitized HI (16–21). A previous piglet study and several rat studies found that TH failed to counteract the increased pathology by measures of magnetic resonance spectroscopy (MRS), amplitude-integrated electroencephalography (aEEG), neuronal cell death, and inflammatory cellular changes (16–21). TH may partly exert neuroprotection by affecting the immune system by suppressing proinflammatory cytokines and the activation of microglia in the brain (22). However, this immunomodulation could be altered when several inflammatory exposures are present simultaneous (23). As both infection and HI may be involved in the pathology of neonatal encephalopathy, several newborns may receive an ineffective or even deleterious treatment (24–26).

With exception of one other piglet study, current preclinical evidence on TH following inflammation-sensitized HI is based on rodent studies (16–21, 27). Thus, further studies in large animals are needed to fully evaluate the clinical practice of TH in newborns with moderate to severe encephalopathy in the context of infection. We aimed to investigate the neuroprotective effect of TH in newborn piglets following LPS-sensitized HI assessed by brain MRS and magnetic resonance imaging (MRI), aEEG, cerebral immunohistochemical markers, and concentrations of blood cells and cytokines.

## 2 Methods

### 2.1. Ethical statement

This study was approved by the Danish Animal Experiments Inspectorate (2019-15-0201-00232) and reported according to the Animal Research: Reporting of *In Vivo* Experiments (ARRIVE) guidelines (Supplementary Material S1) (28).

### 2.2. Study design

**Figure 1:**
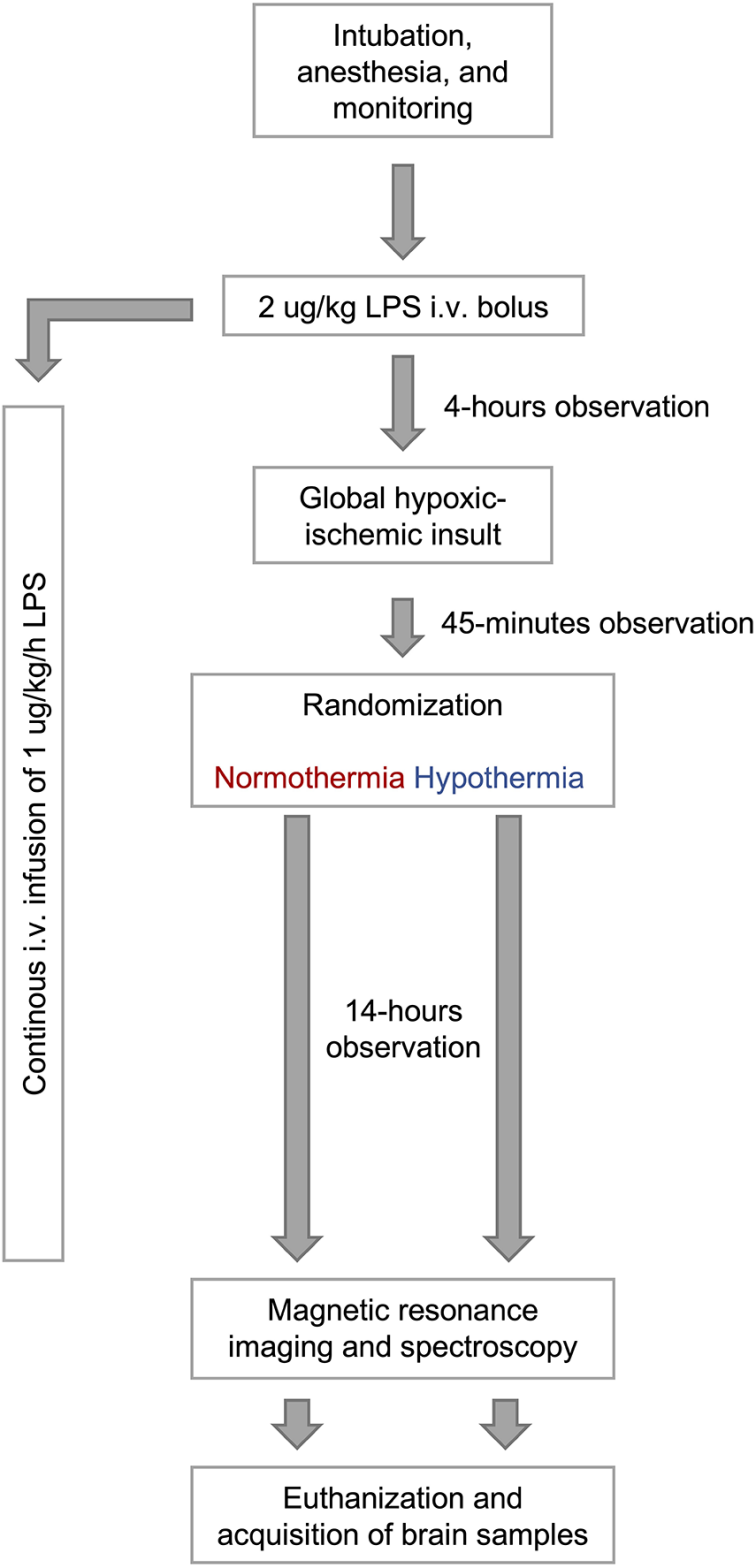
Overview of study investigating the neuroprotective effect of therapeutic hypothermia in newborn piglets with lipopolysaccharide-sensitized hypoxia-ischemia.

A randomized experimental animal study was performed (Figure 1). We included 32 Danish Landrace piglets of either sex, less than 24 h of age, and weighing between 1,200 and 2,300 g. The animals were provided from herds included in a health-monitoring program for slaughter pigs, screened for several pathogens that could affect pigs in a livestock production setting. On each experimental day, two piglets from the same litter were included. An intravenous infusion of LPS was initiated. After 4 h, the animals were exposed to global HI. One animal was then randomized to either normothermia (NT group) or hypothermia (TH group), while the sibling was allocated to the opposite group. The animals were then observed for 14 h with continuous aEEG before undergoing MRS and MRI. Our primary outcome was the thalamic lactate/N-acetylaspartate (Lac/NAA) ratio. After MR-scanning, the animals were euthanized by 80 mg/kg pentobarbital (Richter Pharma, Austria) and brain samples were acquired for immunohistochemical analyses. Two sham piglets—not subjected to LPS-infusion or hypoxia-ischemia—were also investigated with immunohistochemistry in an attempt to establish normal values. Blood samples were taken repeatedly during the experiments for analyses of blood cells and cytokines.

### 2.3. Experimental procedures

#### 2.3.1. Anesthesia and monitoring

The animals were anesthetized by inhalation of 3%–4% sevoflurane (AbbVie, USA). Before endotracheal intubation, the animals were intravenously injected with 5 mg/kg propofol (Fresenius Kabi, Germany), 10 μg/kg fentanyl (B. Braun, Germany), and 1 mg/kg rocuronium (Hameln Pharma, Netherlands). Standard ventilator settings included fraction of inspired oxygen (FiO_2_) = 21%, respiratory frequency (RF) = 25/min, and peak inspiratory pressure = 15 cmH_2_O. These settings were continuously adjusted to maintain a saturation above 93% and end-tidal CO_2_ between 4.5 and 5.5 kPa. Anesthesia was initiated by intravenous infusion of 4–12 mg/kg/h propofol and 5–10 μg/kg/h fentanyl. To maintain hydration and glucose levels, the animals were given 10 ml/kg/h NeoKNaG (15 mmol/L Na^+^, 25 mmol/L Cl^−^, 10 mmol/L K^+^, 505 mmol/L glucose). Catheters were inserted in the umbilical artery and vein to monitor blood pressure, obtain blood samples, and administer drugs. Arterial blood gas samples were analyzed regularly for gas composition, electrolytes, glucose, lactate, and pH-values (ABL Radiometer Medical Denmark). Prophylactic antibiotic treatment was given once with 30 mg/kg ampicillin (Stada, Germany) and 5 mg/kg gentamycin (B. Braun, Germany). The rectal temperature was measured and maintained within the physiological range (38.5–39.5°C) by an air heated mattress. Electrodes for electrocardiography were placed to observe heart rate and rhythms.

#### 2.3.2. Lipopolysaccharides

A bolus of 2 μg/kg LPS (serotype 055-B5) from *Escherichia coli* (Sigma, Germany) was given intravenously followed by continuous infusion of 1 μg/kg/h until MR-scanning.

#### 2.3.3. Hypoxia and ischemia

Infusion of anesthetics and fluid was reduced to half the initial dose before HI to minimize accumulation and continued onward. A single-channel aEEG was attached to the scalp of the piglets to monitor brain activity (Niculet Monitor, Natus Medical Incorporated, USA). The HI insult was induced according to our protocol (29). FiO_2_ was reduced to 4% with RF of 16/min to obtain an upper trace of aEEG <7 μV. FiO_2_ was then adjusted to maintain the upper trace <7 μV for as long as possible within a 45-min period with a mean arterial blood pressure (MABP) <70% of baseline for 10 min. Resuscitation was initiated with FiO_2_ of 21% and RF of 30/min followed by titration to maintain adequate saturation.

#### 2.3.4. Therapeutic hypothermia

Whole-body TH was initiated 45 min after HI with cooling elements until reaching target temperature of 33–34°C and then continued until euthanasia.

#### 2.3.5. Hypotension

Hypotension was treated stepwise by (1) reducing infusion of propofol and fentanyl while still maintaining adequate sedation, (2) bolus infusion of 10 ml/kg isotonic NaCl solution, and (3) infusion of inotropies including 0.25–1.5 μg/kg/min norepinephrine (Macure Pharma, Denmark), 5–15 μg/kg/min dobutamine (Stada, Germany), 0.1–1.5 μg/kg/min epinephrine (Bradex S.A., Greece), and/or 2.5 mg/kg methylprednisolone (Solu-Medrol, Pfizer, USA). If these treatments were unable to maintain appropriate MABP, the animal was euthanized.

### 2.4. Experimental outcomes

#### 2.4.1. Magnetic resonance spectroscopy and imaging

MRS and MRI were conducted as previously described with few adjustments (30, 31). Single voxel proton MRS (TR/TE 2,000/288 ms, 1,024 sample points, spectral width 1,200 Hz, 128 averages) was obtained from the thalamus (10 × 10 × 15 mm), frontal cortex (8 × 8 × 8 mm), occipital cortex (8 × 8 × 8 mm), and white matter (8 × 8 × 12 mm) using point-resolved spectroscopy (PRESS) acquisition (Supplementary Material S2). N-acetylaspartate (NAA, 2.02 ppm), lactate (Lac, 1.33 ppm), creatine (Cr, 3.02 ppm), and choline (Cho, 3.20 ppm) were identified. MRS data were analyzed with LCModel (Stephen Provencer, Canada) version 6.3-1l. MRS outcomes included Lac/NAA, NAA/Cr, and NAA/Cho ratios. Lac/NAA ratios were log-transformed to normalize the values. When lactate was below the detection level, the value was set to half the lowest value among the other piglets. The apparent diffusion coefficient (ADC) was calculated from diffusion weighted images by single-exponential fitting. Blood oxygen level dependent (BOLD) signals were obtained from T2*-maps acquired by multiecho gradient echo sequences by single-exponential fitting. ADC and BOLD images of the right thalamus were analyzed with Horos software (Annapolis, MD, USA) version 3.3.5. Cerebral blood flow was assessed by arterial spin-labelling (ASL) by subtracting control and label images and multiplying with the proper scaling factor. Whole-brain perfusion was calculated in three slices and averaged (Supplementary Material S3). Neuroimaging data were analyzed blinded to randomization.

#### 2.4.2. Amplitude-integrated electroencephalography

aEEG was recorded continuously following HI. However, only one of the two animals on each experimental day was randomized to aEEG recording due to equipment limitations. Points were awarded for different patterns as previously described (32, 33); 0 points for flat trace, 1 point for continuous low voltage, 2 points for burst suppression, 3 points for discontinuous normal voltage, and 4 points for continuous normal voltage. Recordings were analyzed for each hour and then averaged for 0–7 h and 8–14 h. Seizures were identified clinically and based on the aEEG patterns (34). The aEEG recordings were reviewed and analyzed blinded to randomization.

#### 2.4.3. Immunohistochemistry

After euthanasia, the right cerebral hemisphere was immersed in 4% formaldehyde for 7 days at 4°C. Each sample was dissected into coronal slices of 5 mm for dehydration and paraffin embedding. Sections were realized at 5 µm with a microtome. The primary antibodies included anti-Glial fibrillary acidic protein (GFAP, DAKO Z334, 1:1,000), anti-ionizing adaptor protein 1 (IBA1, Wako 019-19741; 1:500), and cleaved-caspase 3 antibody (CC3 Cell Signaling 9661; 1:1,500)—carried out on a Leica™ Bond-Max automat according to protocols routinely used (Bond Polymer Refine IHC protocol F). Sections were counterstained with Nissl labeling. For each immunohistochemical labeling, three slices (frontal, parietal, and occipital) per animal were scanned with NanoZoomer (Hamamatsu, Japan) at a magnification corresponding to 20× objective. For each slice, optical density was calculated from grayscale images standardized to the photomicrograph background as previously described by McAdams et al. (35) and Rangon et al. (36). For each animal, one measurement (1 mm^2^) in each region (thalamus, periventricular white matter, parietal cortex, caudate nucleus, putamen, capsula interna, capsula externa, and hippocampus) was made. For GFAP and IBA1 labeling, the intensity was calculated by densitometric analysis using the NIH ImageJ software (Fiji, NIH). For CC3 labeling, the number of cells per region was reported using the NIH ImageJ software (Fiji, NIH).

#### 2.4.4. Blood cells and cytokines

Arterial blood samples were collected at baseline, 3 h after initiation of LPS infusion, immediately after HI, 6 h after HI, and 12 h after HI. The samples were analyzed by a veterinary hematology analyzer (ProCyte Dx, IDEXX, USA) to assess the concentration of red blood cells, platelets, total white blood cells, neutrophils, lymphocytes, and monocytes. Samples taken after HI were also centrifugated at 3,163 g at 18°C for 10 min and plasma was transferred to cryotubes and stored at −80°C for later assessment of cytokines. Cytokines were assessed with the Cytokine and Chemokine 9-plex Porcine ProcartaPlex (ThermoFisher, USA) including IL-1β, IL-4, IL-6, IL-8, IL-10, IL-12p40, IFN-α, IFN-γ, and TNF-α. Samples were measured in duplicates and analyzed using the Bio-Plex Manager 6.0 Software (BioRad, USA). If cytokines were below the detection level, they were replaced by half the detection limit.

### 2.5. Sample size

As our primary outcome was the thalamic Lac/NAA ratio, the sample size was based on a previous piglet study finding a difference in Lac/NAA ratio after 9.5 h between piglets with HI treated with and without TH (1.83 ± 0.31 vs. 2.35 ± 0.49) (37). When comparing the means provided by this study, 12 piglets should be included in each group to show a difference with a two-sided statistical significance level of 5% and a power of 80%. Based on previous studies by our group, we expected mortality of around 20% and therefore included 16 piglets in each group (38, 39).

### 2.6. Statistical methods

Independent and continuous data were analyzed by unpaired *t*-test (normally distributed) or Mann–Whitney test (non-normally distributed), while dependent data were analyzed by paired *t*-test. Categorical data were analyzed by Fisher's exact test. Outcomes with repeated measures were analyzed by mixed-effects analyses with assumed sphericity and randomly missing values followed by Fisher's LSD test. Normally distributed data are presented as mean values with standard deviations, while non-normally distributed data are presented as median values with interquartile ranges. Differences with a two-sided *p*-value less than 0.05 were considered statistically significant.

As blood glucose in piglets treated with TH was more than twice the value of that in the normothermic piglets before scanning, *post-hoc* multivariate linear regression was conducted with adjustment of these values for Lac/NAA ratios and immunohistochemical markers in the thalamus, white matter, and cortex*.* Statistical analyses were performed by GraphPad Prism version 8.0.0 for MacOS (GraphPad Software, San Diego, California USA, www.graphpad.com).

## 3. Results

### 3.1. Animal characteristics

A total of 15 female and 17 male piglets were included (NT = 7/9, TH = 8/8). We observed no difference in weight between piglets treated with and without TH (1,650 g vs. 1,775 g). The severity of the HI insult was similar between our two groups based on duration of aEEG <7 μV, duration of MABP <70% of baseline, and end-hypoxia pH and lactate (Table 1). Difficulties with titrating FiO_2_ to secure target MABP while still keeping the piglets alive during the HI insult resulted in durations of target MABP below the aim of 10 min. The piglets received similar total dose of anesthetics and inotropies (Supplementary Material S4). Vital signs and arterial blood values were similar between groups before LPS infusion and HI. At the end of observation, piglets treated with TH had lower temperature, heart rate, and p-sodium, but higher p-glucose and p-lactate (Table 2).

**Table 1:** The severity of the hypoxic-ischemic insult in newborn piglets following lipopolysaccharide-sensitization.

** **	NT group	TH group	*p*-value
aEEG <7** **μV (min)	39 (32–44)	41 (40–43)	0.13
aEEG <5** **μV (min)	27 (13.6)	29 (8.7)	0.60
Target MABP (mmHg)	39 (35–42)	39 (33–41)	0.86
MABP <70% baseline (min)	5 (1.3–8.0)	3.5 (0.0–6.8)	0.80
Lowest MABP (mmHg)	31 (12.1)	32 (10.8)	0.79
End-hypoxia pH	6.76 (6.75–6.84)	6.83 (6.75–6.88)	0.26
End-hypoxia lactate (mmol/L)	18.7 (3.9)	19.2 (4.1)	0.75

Values are compared between piglets treated without (NT) and with therapeutic hypothermia (TH). Normally distributed data were analyzed by unpaired *t*-test and presented as mean values with standard deviations, while non-normally distributed data were analyzed by Mann–Whitney test and presented as median values with interquartile ranges.

**Table 2:** Vital and arterial blood-gas values in newborn piglets subjected to lipopolysaccharide-sensitized hypoxia-ischemia.

	LPS baseline	HI baseline	HI insult^1^	30 min	1 h	3 h	6 h	Before scans
MABP (mmHg)								
NT group	54 (6.3)	54 (6.0)		53 (11.8)	48 (9.4)	42 (11.1)	45 (56–40)	46 (9.5)
TH group	53 (8.6)	53 (9.5)		48 (14.4)	44 (8.1)	47 (6.9)	50 (56–46)	45 (16.3)
Heart rate (bpm)								
NT group	153 (26.7)	179 (29.5)		194 (26.1)	214 (20.8)	225 (35.1)	216 (43.5)	201 (48.6)
TH group	155 (35.9)	186 (25.5)		193 (16.6)	**192** (34.6)*	**169** (21.1)*	**180** (27.6)*	**164** (31.8)*
Temperature (°C)								
NT group	38.9 (38.6–39.3)	39.2 (0.3)		38.3 (0.5)	38.6 (0.7)	39.0 (0.6)	39.0 (0.4)	38.8 (0.4)
TH group	39.0 (38.5–39.4)	39.2 (0.3)		38.1 (0.7)	**36.8** (1.7)*	**33.8** (0.6)*	**33.8** (0.7)*	**33.7** (0.7)*
eCO_2_ (kPa)								
NT group	5.8 (0.8)	6.1 (0.7)		6.3 (1.2)	5.8 (1.0)	5.6 (1.1)	5.7 (1.1)	6.0 (1.9)
TH group	5.8 (0.9)	5.9 (1.0)		**5.2** (1.0)*	**5.0** (0.9)*	5.5 (1.4)	5.5 (1.5)	5.2 (1.5)
pO_2_ (kPa)								
NT group	11.7 (9.8–14.2)	11.1 (3.8)		21.0 (15.2–32.6)	16.5 (14.0–28.4)	22.3 (13.1)	20.5 (6.9)	18.5 (10.1)
TH group	11.1 (9.3–14.3)	11.5 (2.4)		17.9 (15.7–27.7)	17.6 (13.6–22.0)	28.6 (16.3)	22.9 (9.1)	23.2 (11.3)
pH								
NT group	7.51 (7.46–7.56)	7.45 (0.07)		6.95 (0.14)	7.12 (0.16)	7.30 (0.20)	7.41 (0.19)	7.39 (0.15)
TH group	7.48 (7.45–7.57)	7.47 (0.07)		6.96 (0.15)	7.12 (0.17)	7.20 (0.13)	7.27 (0.10)	7.29 (0.17)
Lactate (mmol/L)								
NT group	1.2 (0.4)	1.4 (1.8–1.0)		14.7 (3.0)	11.2 (3.2)	3.8 (2.5–8.5)	2.8 (2.1)	1.8 (0.7–2.3)
TH group	1.3 (0.5)	1.6 (1.8–1.1)		14.6 (3.0)	12.0 (3.6)	5.0 (4.5–8.2)	3.2 (2.0)	**3.2** (2.7–4.5)*
Base excess (mmol/L)								
NT group	7.1 (4.4–10.1)	3.2 (4.1)		−22.3 (4.8)	−17.1 (6.5)	−7.2 (8.8)	−3.2 (7.8)	−0.3 (6.0)
TH group	5.1 (2.4–7.3)	2.2 (3.9)		−23.2 (5.5)	−17.3 (5.8)	−10.3 (7.2)	−6.0 (6.4)	−6.3 (9.5)
Glucose (mmol/L)								
NT group	6.7 (4.9–8.7)	5.1 (4.4–5.2)		10.4 (3.1)	8.5 (3.2)	5.9 (2.7)	5.0 (1.8)	4.5 (1.2)
TH group	7.0 (5.7–8.0)	5.2 (4.3–6.0)		9.9 (2.7)	9.0 (3.5)	**9.6** (5.4)*	8.2 (4.8)	**9.4** (5.7)*
Sodium (mmol/L)								
NT group	138 (137–140)	136 (2.0)		138 (2.5)	137 (2.2)	135 (1.6)	134 (2.5)	132 (3.3)
TH group	137 (135–141)	136 (2.8)		137 (3.0)	136 (2.8)	**134** (2.6)*	132 (3.8)	**129** (4.0)*
Potassium (mmol/L)								
NT group	2.9 (0.7)	2.9 (0.6)		2.9 (0.6)	3.5 (0.5)	3.7 (0.9)	4.2 (0.9)	4.7 (1.1)
TH group	2.9 (0.6)	2.9 (0.7)		2.9 (0.7)	3.1 (0.9)	3.8 (1.1)	4.2 (1.1)	4.9 (1.2)

LPS, lipopolysaccharide; HI, hypoxia-ischemia; MABP, mean arterial blood pressure.

Values are compared between piglets treated without (NT) and with therapeutic hypothermia (TH). Normally distributed data were analyzed by unpaired *t*-test and presented as mean values with standard deviation, while non-normally distributed data were analyzed by Mann–Whitney test and presented as median values with interquartile range.

Bold values emphasize statistical significance.

* *p*-value < 0.05.

^1^ The HI insult severity is presented in Table 1.

### 3.2. Survival and missing information

A total of 12 piglets died during the experiments due to refractory hypotension (NT = 6, TH = 6). A total of 18 piglets were scanned (NT = 9, TH = 9). One piglet in each group survived the observation period but could not be scanned due to logistic difficulties. Technical issues resulted in missing MRS data from the frontal and occipital cortex in five piglets, missing ASL data in four piglets, and missing ADC and BOLD data in one piglet. Due to laboratory Covid-19 lockdown, five brain hemispheres were stored in phosphate-buffered saline for one-and-a-half month before embedding. Blood cell analyses were possible in 19 piglets (NT = 10, TH = 9) and cytokine analyses in 23 piglets (NT = 10, TH = 13).

### 3.3. Outcomes

#### 3.3.1. Magnetic resonance spectroscopy and imaging

**Figure 2:**
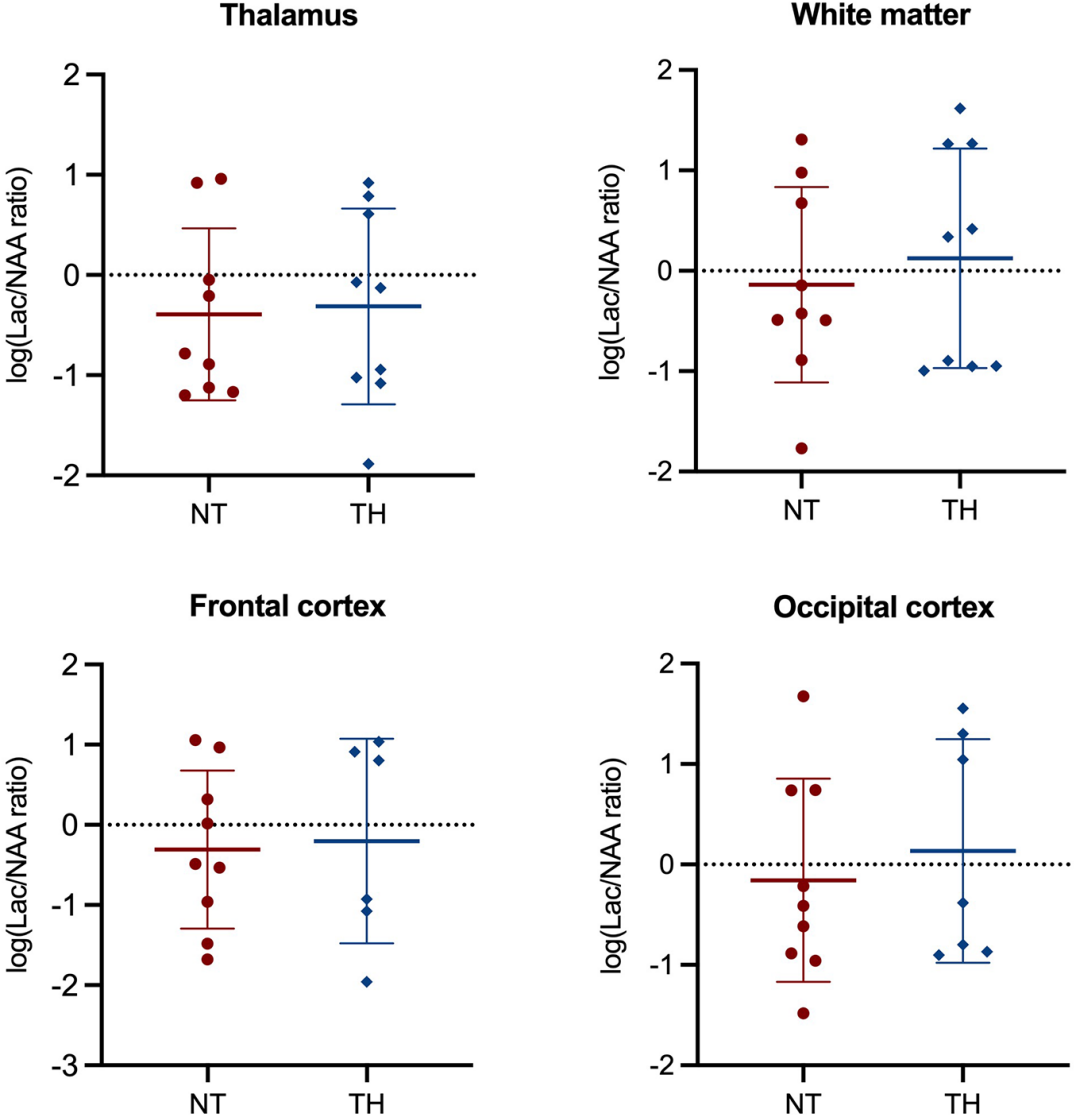
Magnetic resonance spectroscopy used to assess the Lac/NAA ratio in newborn piglets following lipopolysaccharide-sensitized hypoxia-ischemia. Values are compared between piglets treated without (NT) and with therapeutic hypothermia (TH). Data were log-transformed and analyzed by unpaired *t*-test and presented with mean values and standard deviation.

**Figure 3:**
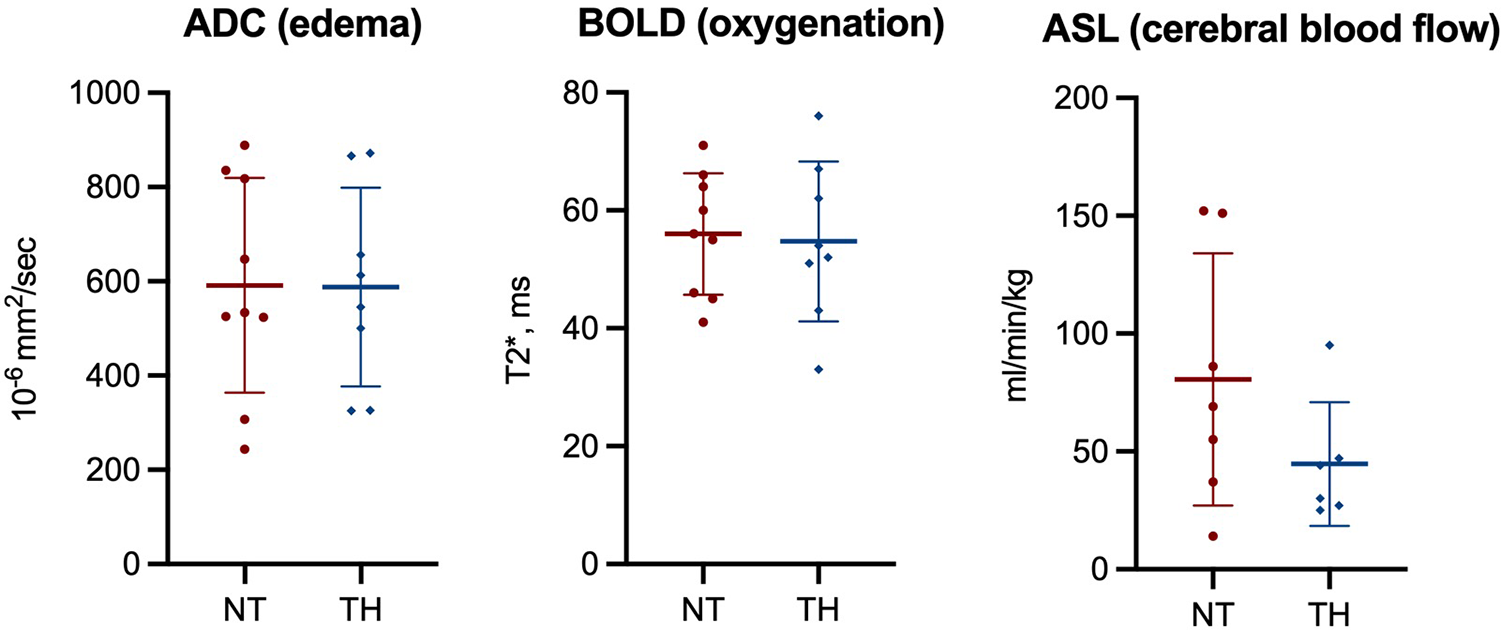
Magnetic resonance imaging used to assess apparent diffusion coefficient (ADC), blood oxygen level dependent (BOLD) signals, and cerebral blood flow (ASL) in piglets following lipopolysaccharide-sensitized hypoxia-ischemia. Values are compared between piglets treated without (NT) and with therapeutic hypothermia (TH). Data were analyzed by unpaired *t*-test and presented with mean values and standard deviation.

We found no statistically significant difference in Lac/NAA ratio in any brain region between piglets treated with and without TH (Figure 2). Furthermore, we found no difference in NAA/Cr or NAA/Cho ratios (Supplementary Material S5 and S6). At last, we found no difference in ADC, BOLD, or ASL values between the two groups (Figure 3).

#### 3.3.2. Amplitude-integrated electroencephalography

We found no statistically significant difference between piglets treated with and without TH in mean aEEG score from 1 to 7 h (1.8 vs. 1.6) and 8–14 h (2 vs. 2) (Supplementary Material S7). We observed no statistically significant difference in number of piglets with seizures (NT = 27%, TH = 43%).

#### 3.3.3. Immunohistochemistry

**Figure 4:**
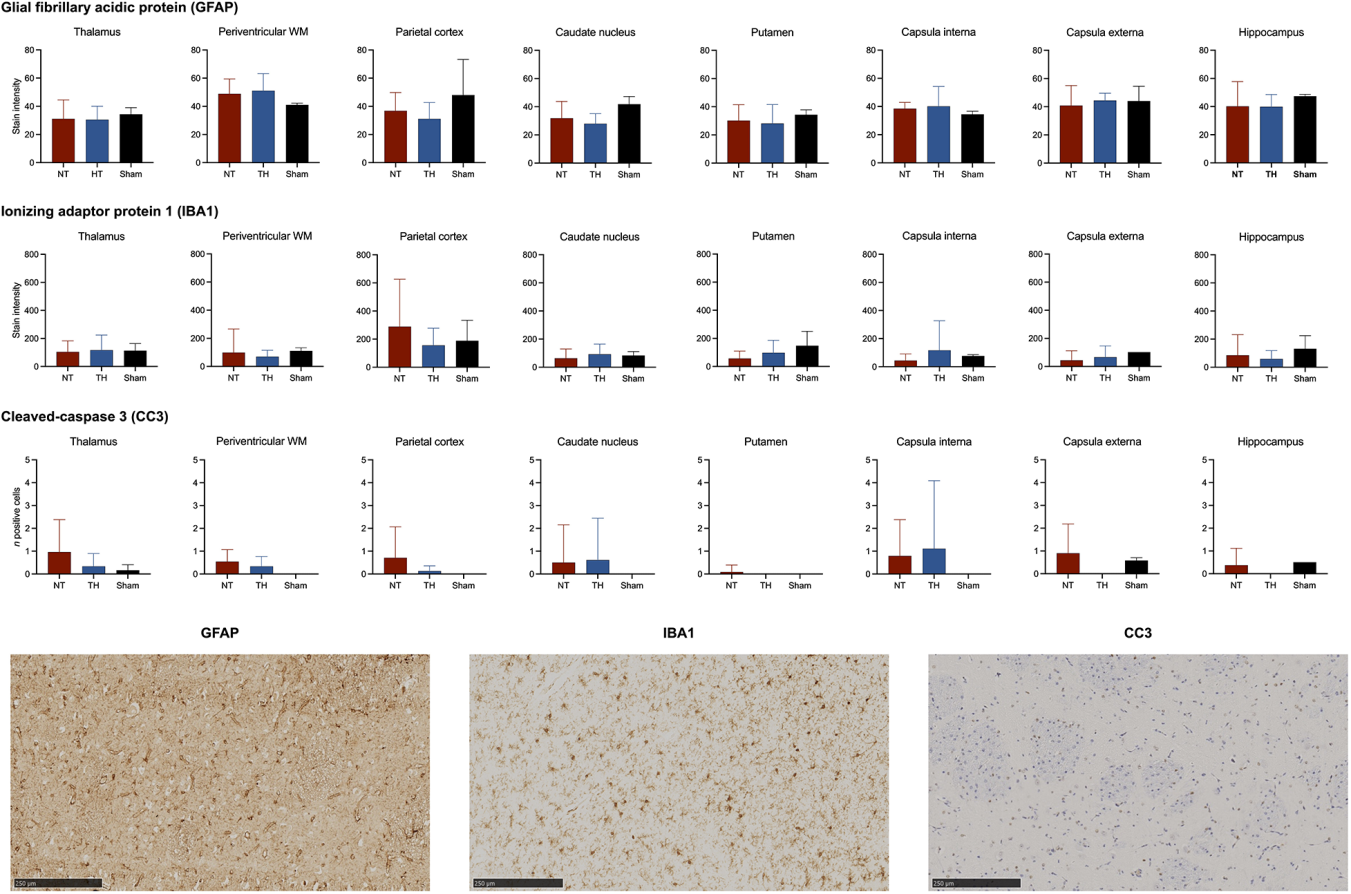
Glial fibrillary acidic protein (GFAP), ionized calcium-binding adaptor molecule (IBA1), and cleaved-caspase 3 (CC3) expressed in piglets following lipopolysaccharide-sensitized hypoxia-ischemia. Values are compared between piglets treated without (NT) and with therapeutic hypothermia (TH). Data were analyzed by unpaired *t*-test and presented with mean values and standard deviation.

Examples of immunohistochemical stainings are provided for each marker (Supplementary Material S8). We found no statistically significant difference in GFAP (density), IBA1 (density), or CC3 (cells/mm^2^) in any brain region between piglets treated with and without TH (Figure 4). The experimental piglets appeared to have similar values as the sham piglets (Figure 4).

#### 3.3.4. Blood cells and cytokines

**Figure 5:**
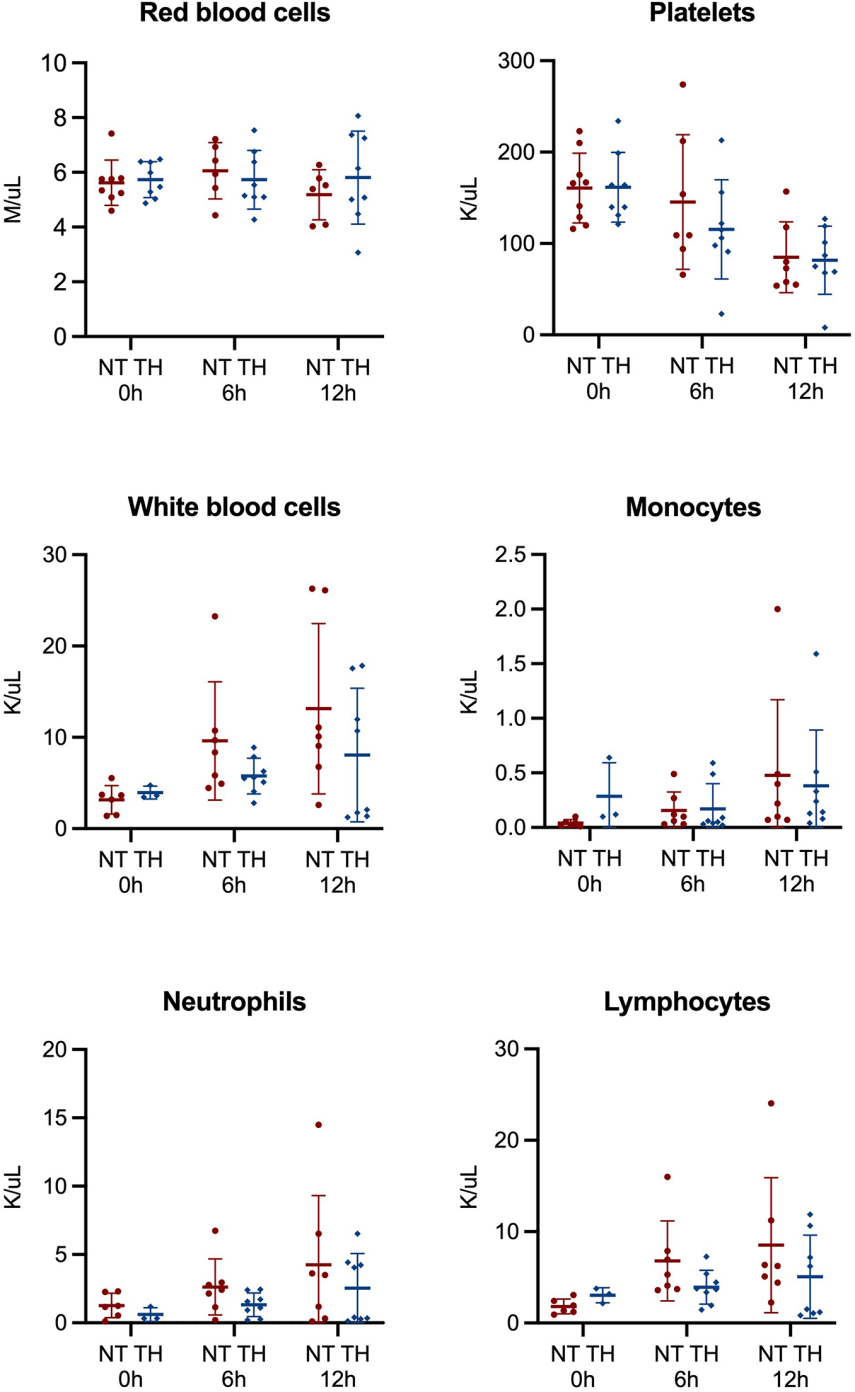
Cells analyzed from blood samples 0, 6, and 12 h in newborn piglets following lipopolysaccharide-sensitized hypoxia-ischemia. Values are compared between piglets treated without (NT) and with therapeutic hypothermia (TH). Data were analyzed by mixed-effect analysis followed by Fisher's LSD test and presented with mean values and standard deviation.

**Figure 6:**
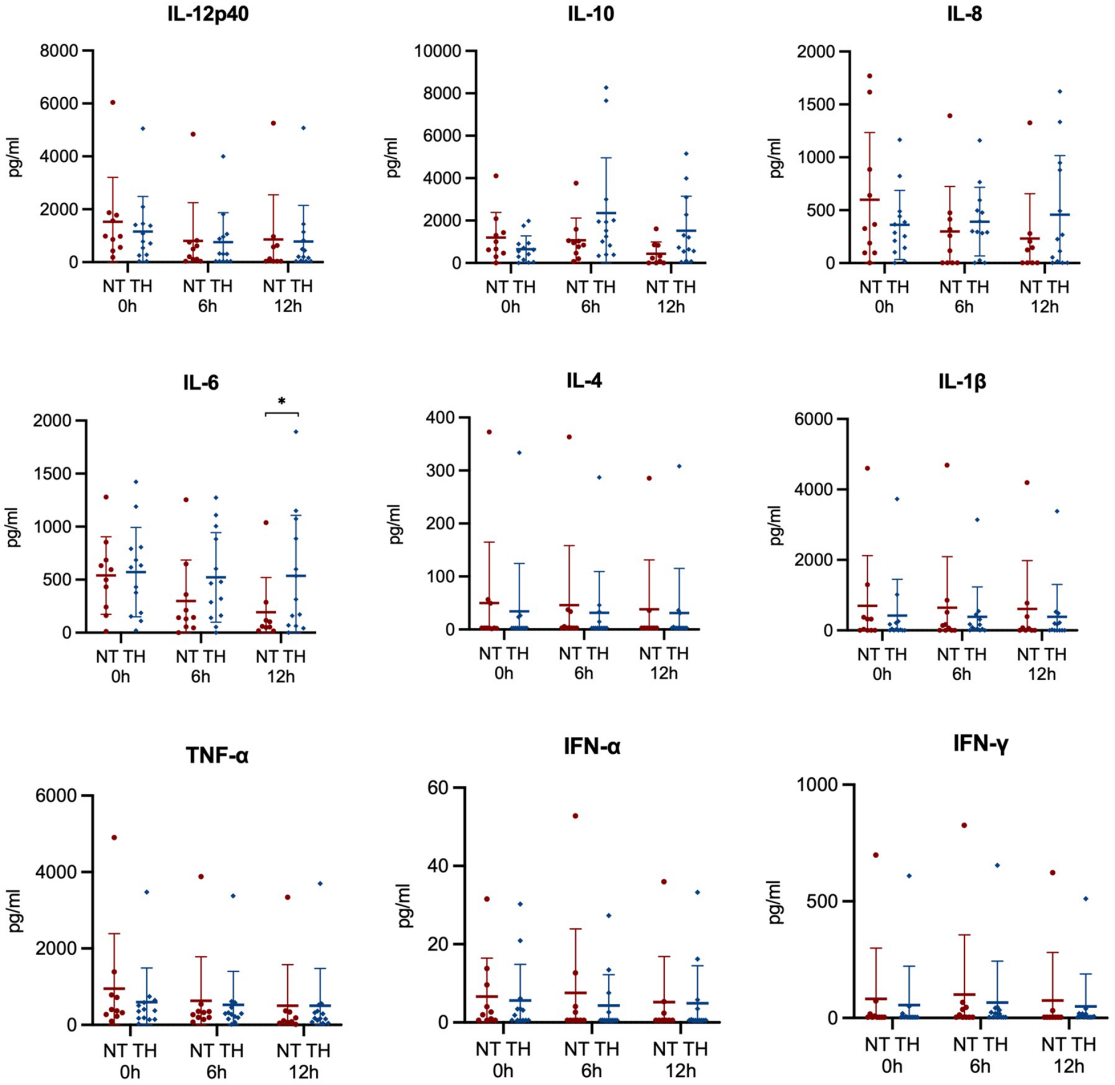
Cytokines analyzed from blood samples 0, 6, and 12 h in newborn piglets following lipopolysaccharide-sensitized hypoxia-ischemia. Values are compared between piglets treated without (NT) and with therapeutic hypothermia (TH). Data were analyzed by mixed-effect analysis followed by Fisher's LSD test and presented with mean values and standard deviations. *p-value < 0.05.

Comparisons of mean blood cells before and three hours after LPS infusion showed increased red blood cell counts (4.9 vs. 5.6 M/μl) and decreased platelets (254 vs. 183 K/μl), white blood cells (9.0 vs. 1.1 K/μl), neutrophils (6.1 vs. 0.4 K/μl), lymphocytes (2.6 vs. 0.7 K/μl), and monocytes (0.21 vs. 0.01 K/μl). Following the HI insult, the concentration of platelets decreased, while the concentration of white blood cells increased. We found no statistically significant difference in concentration of blood cells between piglets treated with and without TH (Figure 5). Similarly, we observed no overall difference in concentrations of cytokines between our two groups (Figure 6). However, we found statistically significant higher mean values of IL-6 after 12 h in piglets treated with TH (170 vs. 535 pg/ml, *p*-value = 0.05). The concentration of IL-6, IL-12p40, and TNF-α decreased over time, while the concentration of IFN- γ increased until the 6-h assessment and then decreased.

#### 3.3.5. Hyperglycemia

Adjustment for blood glucose before scanning failed to reveal any statistically significant difference between piglets treated with and without TH in Lac/NAA ratios and immunohistochemical markers in the thalamus, white matter, and cortex (Supplementary Material S9).

## 4. Discussion

### 4.1. Summary of findings

We investigated the neuroprotective effect of TH following LPS-sensitized HI in a randomized experimental piglet study. We found no indication of early neuroprotection by measures of MRS, MRI, aEEG, immunohistochemistry, and concentration of blood cells and cytokines. The piglet study of Martinello et al. (21) also found TH without neuroprotective effects following LPS-sensitized HI. They investigated similar outcomes at 48 h following rewarming, which may be more appropriate timing for detecting brain injury due to the evolving pathology following HI (40, 41). However, our study adds to their findings as we used a different protocol for global hypoxia-ischemia and included a larger population of piglets with both males and females (42). Our studies may indicate that TH is not neuroprotective when gram-negative infection or inflammation occurs before a HI insult in the human fetus or newborn.

The Lac/NAA ratio is considered one of the most accurate predictors of neurodevelopmental impairment in human newborns with neonatal encephalopathy (43). Furthermore, piglet studies with and without LPS-sensitization have shown the Lac/NAA ratio to correlate with both neuronal cell death and microglia activation at 24 and 48 h after HI (44). Even though the Lac/NAA ratio was pathologically increased in most piglets, we found no differences in Lac/NAA ratio in any brain region in piglets treated with and without TH (43). The Lac/NAA ratio represents secondary energy failure, which is thought to begin 6 to 24 h following HI (40, 41). An effect of TH may accordingly have been revealed if MRS had been conducted at later timepoints rather than at 14 h after HI. However, Martinello et al. (21) found no difference in brain Lac/NAA ratio at 48 h after LPS-sensitized HI.

We used GFAP as a marker of astrogliosis, IBA1 as a marker of microgliosis, and CC3 as a marker of apoptosis. Both astroglia and microglia may induce brain inflammation following LPS exposure as shown *in vitro* (45). This may further lead to activation of apoptotic pathways and possibly cell death (46). Another study found an increased expression of these markers in LPS-sensitized piglets after 48-h observation when compared with piglets subjected to HI alone—indicating an exacerbating effect of LPS-sensitization (15). However, we found no difference in these markers when comparing LPS-sensitized piglets treated with and without TH. As for the Lac/NAA ratio, these findings of no difference may also be explained by our early time of assessment. This is further supported by the lack of difference between the experimental piglets and the two sham piglets. Rodent models have indicated that the expression of GFAP, IBA1, and CC3 may peak at least 24 h after HI (37, 47–49). However, TH has been shown to reduce brain expression of CC3 and TNF-α by Western Blotting already after 6 h in another piglet model of HI (50), while microglial activation may occur already 6 h after initiation of LPS exposure (51). In this study, the total duration of LPS exposure was 19 h. We are unable to rule out an effect of TH at later time points, but other studies with longer observation periods have observed similar findings of no difference (16, 21). The rat study by Osredskar et al. (16) found TH to be unable to counteract the LPS-associated increase in brain expression of GFAP, IBA1, and CC3 with assessments after 24 h and 1 week. Martinello et al. (21) similarly found no difference in brain expression of GFAP and IBA1 at 48 h with exception of an increased IBA1 in the caudate nucleus in normothermic piglets. However, they did find that normothermic piglets had increased expression of CC3 in their whole brain analysis, though with reports of CC3 being a poor marker of neuronal cell death in their model (44). Studies have found TH to suppress both astroglia and microglia activity in animal models of HI alone (52, 53).

To validate the effect of LPS on the immune system, we assessed white blood cells and platelets before and after LPS infusion—finding that LPS was associated with decreased concentration of both cell types. Similar to Martinello et al. (21), we found TH to have no effect on the concentration of these blood cells during our observation period following HI. Clinical studies of neonatal encephalopathy with follow-up from 0 to 7 days of life have found TH to be associated with reduced concentration of white blood cells (54, 55). Though, one study found TH to have no significant effect on white blood cells in newborns with neonatal encephalopathy and sepsis risk factors (54). TH may exert neuroprotection by modulating the production of cytokines (56, 57). Among proinflammatory cytokines, especially TNF-α and IL-1β may be involved in the pathology of LPS-sensitized HI as inhibition of these cytokines seems to eliminate the sensitizing effect of LPS (58–61). We found no difference in any cytokines between piglets treated with and without TH with exception of an increased IL-6 after 12 h in piglets treated with TH. Martinello et al. 21) also found no difference in plasma concentration of TNF-α, while Chevin et al. (18) found no difference in brain expression of both TNF-α and IL-1β in LPS-sensitized newborn rats treated with and without TH. Other piglet studies of HI alone have found TH to reduce the expression of TNF-α in both blood and brain (37, 50, 62).

Together with the current literature, our findings suggest that hypothermia may be unable to suppress the immune system when several inflammatory exposures are present in newborns (16, 21). Immunomodulation has previously been suggested as one of the neuroprotective mechanisms of TH, which thereby indicates that other treatments may be of value in these situations (22). A previous rat study by Bark et al. (63) investigated the neuroprotective effect of azithromycin in newborn rats subjected to HI sensitized by either LPS or Pam_3_Cys-Ser-(Lys)_4_. They found that azithromycin was associated with improved sensorimotor function, brain tissue preservation, and survival at 14 and 28 days after the insult. This may deserve further investigation in larger animal models to thoroughly evaluate its potential.

### 4.2. Limitations and translation

We present a piglet model with both male and female animals subjected to a standardized global HI insult. The severity of the insult was similar between piglets treated with and without TH as indicated by the duration of aEEG and MABP suppression during the insult as well as pH and p-lactate immediately after the insult. Furthermore, several outcomes were evaluated including both brain imaging, electroencephalography, cerebral immunohistochemical markers, and the systemic effect by blood cells and cytokines. However, this study also has some limitations. As mentioned, our markers of brain injury may have differed had they been assessed closer to the secondary phase of injury at 48–72 h (40, 41). This severely limits a statement of no neuroprotective effect by TH in this model. Though, other piglet studies have revealed neuroprotective effects of TH following HI alone with assessments between 6 and 12 h after the insult (37, 50). Additionally, this study could have benefitted from other markers of cell death including TUNEL-staining. Other piglet studies have found high correlation between the Lac/NAA ratio at 24 and 48 h and cell death evaluated by TUNEL-staining at 48 h (44). Another study in newborn piglets found LPS sensitization to exacerbate brain injuries following HI at 48 h (15). We did not compare markers of brain injury between piglets subjected to HI with and without LPS-sensitization. However, similar doses were used as the previous study and the systemic effect of LPS was validated by changes in blood cells following the infusion (21). The clinical translation may also have some limitations. In a recent systematic review, we found similar neurological outcome in newborns with neonatal encephalopathy treated with TH with and without markers of perinatal infection, though based on very low quality of evidence (64). Clinical populations are often more heterogenous than animals in experimental settings and several factors may affect the interaction between the different etiologies and treatments. LPS sensitization has actually shown neuroprotective effects when initiated 24 h before HI (10, 65), indicating that both a negative and positive conditioning may occur in the newborn brain (66). Furthermore, the effect of TH seems to vary according to pathogen. Studies in newborn rats have found neuroprotection by TH following HI sensitized by gram-positive endotoxins (20, 27). These differences may be explained by the different properties of endotoxins from gram-negative and gram-positive bacteria with the earlier activating toll-like-receptors 4 and the later activating toll-like receptors 2 (67, 68). However, the neuroprotective effects of TH following gram-positive sensitization are yet to be investigated in larger animal models. At last, the neuroprotective effect of TH following LPS-sensitized HI has to be considered with regards to gestational ages (69). Studies in newborn rats have investigated LPS sensitization in a wide variety of ages including postnatal day 1 (60, 70, 71), postnatal day 7–8 (61, 72–79), and postnatal day 12 (58, 59), somewhat corresponding to the brain development in preterm-, early term-, and full term human newborns (80). Studies in rats at postnatal day 12 have found TH with some neuroprotective effects following LPS-sensitized HI, showing reduced penumbra injuries and increased levels of antioxidant enzymes (18, 19). In our piglet study, the brain development similarly corresponds to what is seen in full term human newborns (81).

## 5. Conclusion

We found no difference between piglets with LPS-sensitized HI treated with and without TH based on measures of MRS, MRI, aEEG, immunohistochemistry, and concentration of blood cells and cytokines. However, our results may be limited by our early time of assessment of the brain. This may nonetheless indicate that other treatments than TH may be of value in newborns exposed to gram-negative infection or inflammation before a HI insult.

## Data Availability

The original contributions presented in the study are included in the article/**Supplementary Material**, further inquiries can be directed to the corresponding author.
