# Supplementary material for: No neuroprotective effect of therapeutic hypothermia following lipopolysaccharide-sensitized hypoxia-ischemia: a newborn piglet study
Source: Front Pediatr. 2023 Nov 28;11:1268237. doi: 10.3389/fped.2023.1268237 (PMC10715312; doi:10.3389/fped.2023.1268237)
Supplement: Supplementary file 1 [file Datasheet1.docx]

***Supplementary material***

**Title**

No neuroprotective effect of therapeutic hypothermia following lipopolysaccharide-sensitized hypoxia-ischemia: a study in newborn piglets

**Authors**

Mads Andersen^1^, Hannah B. Andersen^1^, Ted C. K. Andelius^1^, Lærke H. Hansen^1^, Regitze Pinnerup^1^, Mette Bjerre^2^, Steffen Ringgard^3^, Leslie Schwendimann^4^, Pierre Gressens^4^, Kasper J. Kyng^1^, Tine B. Henriksen^1^

**Affiliations**

^1^ Department of Paediatrics and Adolescent Medicine, Aarhus University Hospital, Aarhus, Denmark

^2^ Medical Research Laboratory, Aarhus University Hospital, Aarhus, Denmark

^3^ MR Research Centre, Aarhus University Hospital, Aarhus, Denmark

^4^ NeuroDiderot, Inserm, Université Paris Cité, Paris, France

**Data access**

Data access may be provided by contact to corresponding author.

**Protocol registration**

The protocol for the study was designed *a priori*, but was not registered in any public databases.

**Supplementary 1. Animal Research: Reporting of In Vivo Experiments (ARRIVE) guidelines**

**
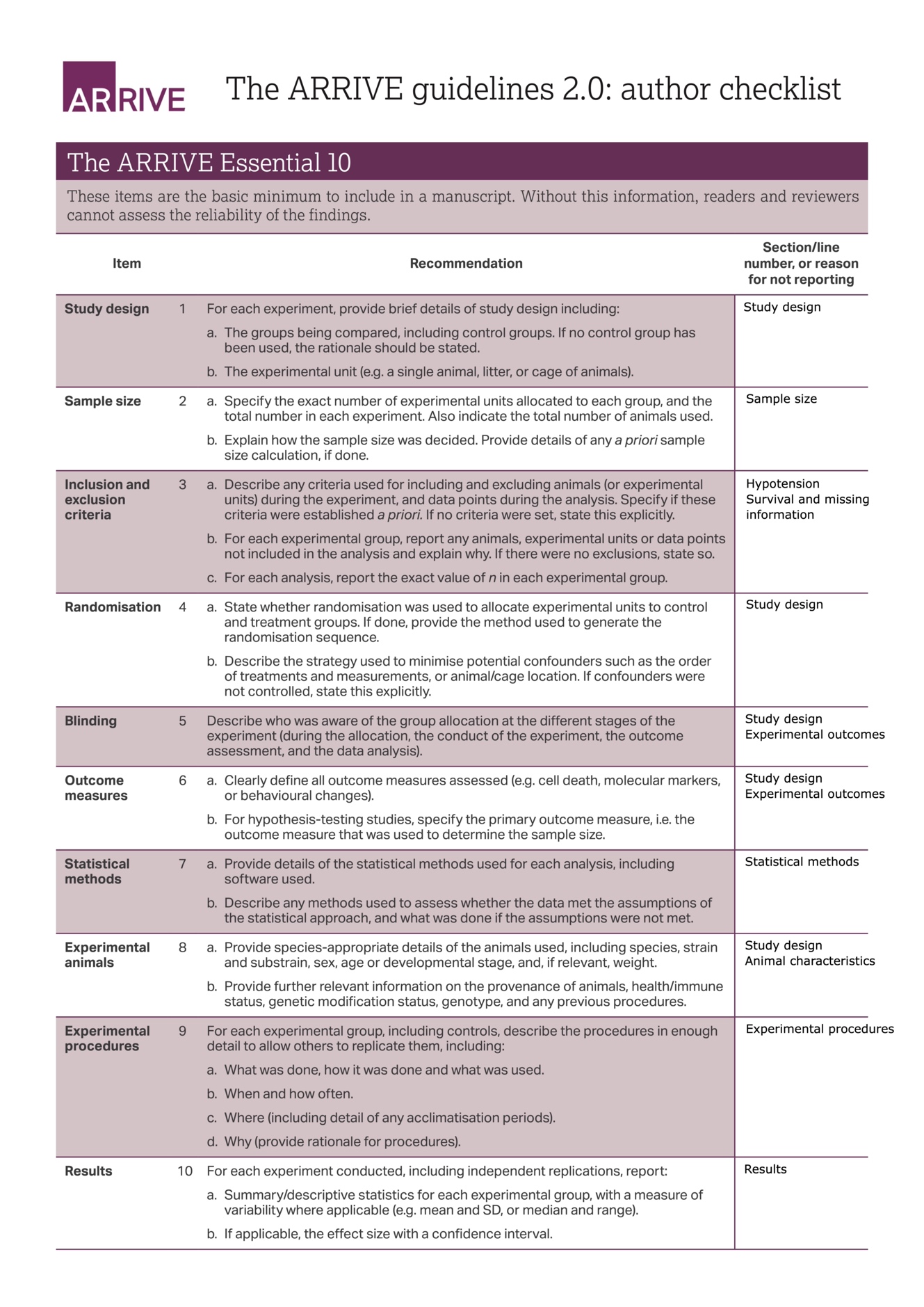
**

**
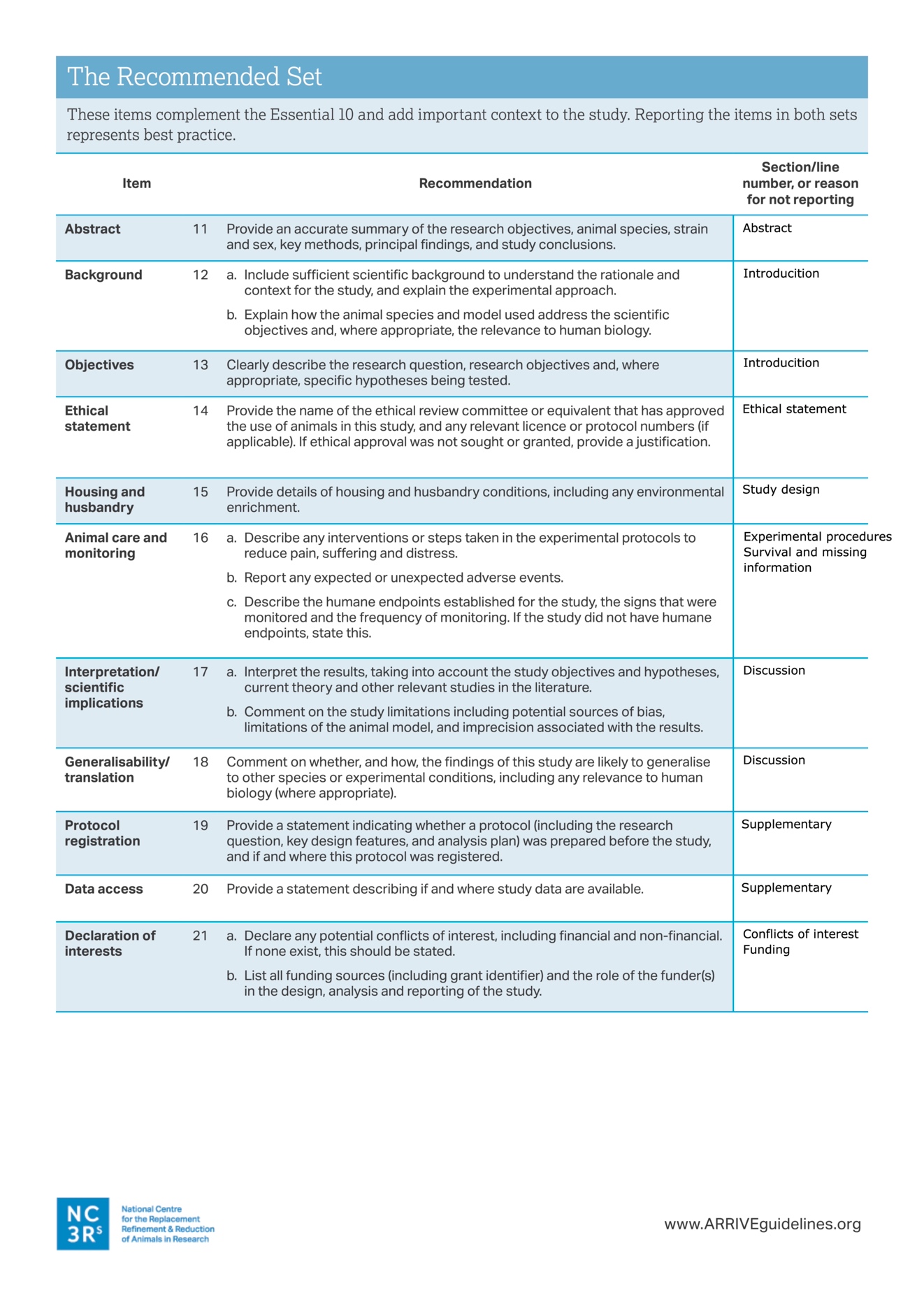
**

**Supplementary 2. Voxel placement during magnetic resonance spectroscopy in newborn piglets following lipopolysaccharide-sensitized hypoxic-ischemia.**

**
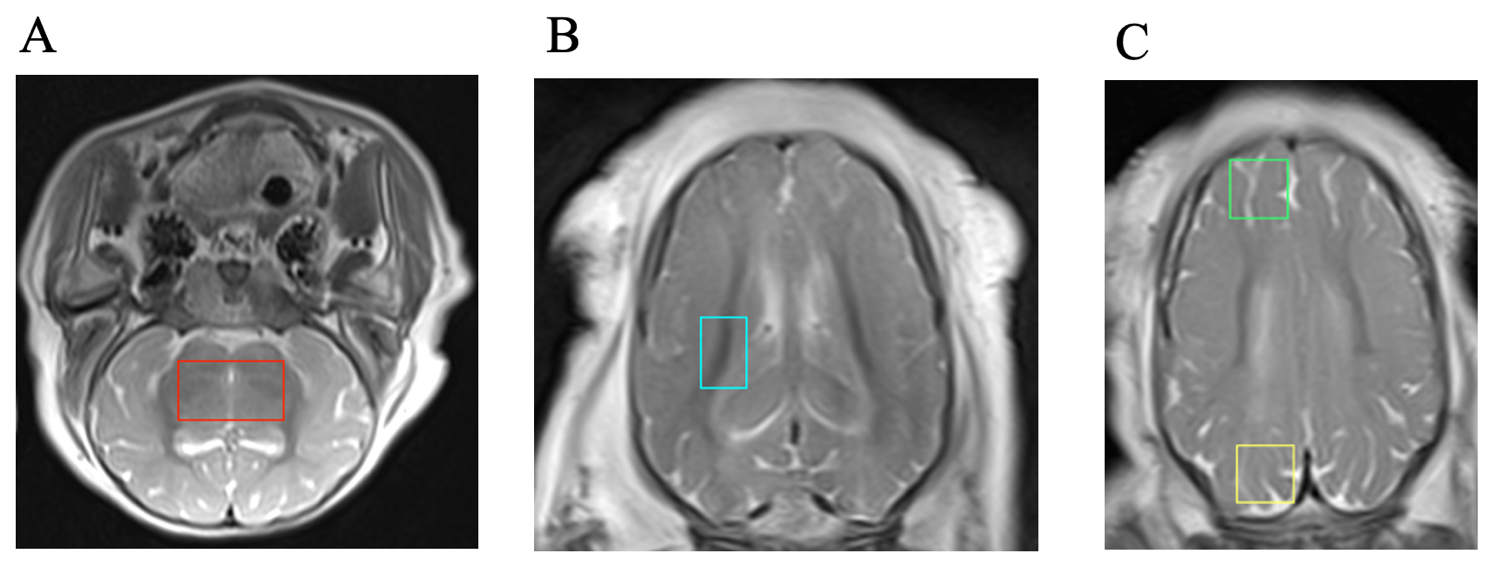
**

A) thalamus in the axial plane, B) white matter, C) frontal and occipital cortex.

**Supplementary 3. Magnetic resonance imaging sequences variables, orientation, and regions of interest.**

| **Sequence** | **Type** | **Orientation** | **TR (ms)** | **TE (ms)** | **Slice thickness (mm)** | **Matrix** | **FOV (mm^2^)** |
| --- | --- | --- | --- | --- | --- | --- | --- |
| T2-weigthed | Fast spin-echo | Axial/coronal | 6430 | 74 | 2 | 320x240 | 160x160 |
| Diffusion-weighted^1^ | Single shot EPI | Coronal | 3300 | 108 | 3 | 196x190 | 213x206 |
| T2*-maps^2^ | Multi-echo gradient-echo | Coronal | 431 | 3.67-49 | 4 | 192x126 | 180x118 |

TR, repletion time; TE, echo time; FOV, field of view.

^1^ The b-value was 800 s/mm^2^.

^2^ Sequence was completed with 11 echoes.


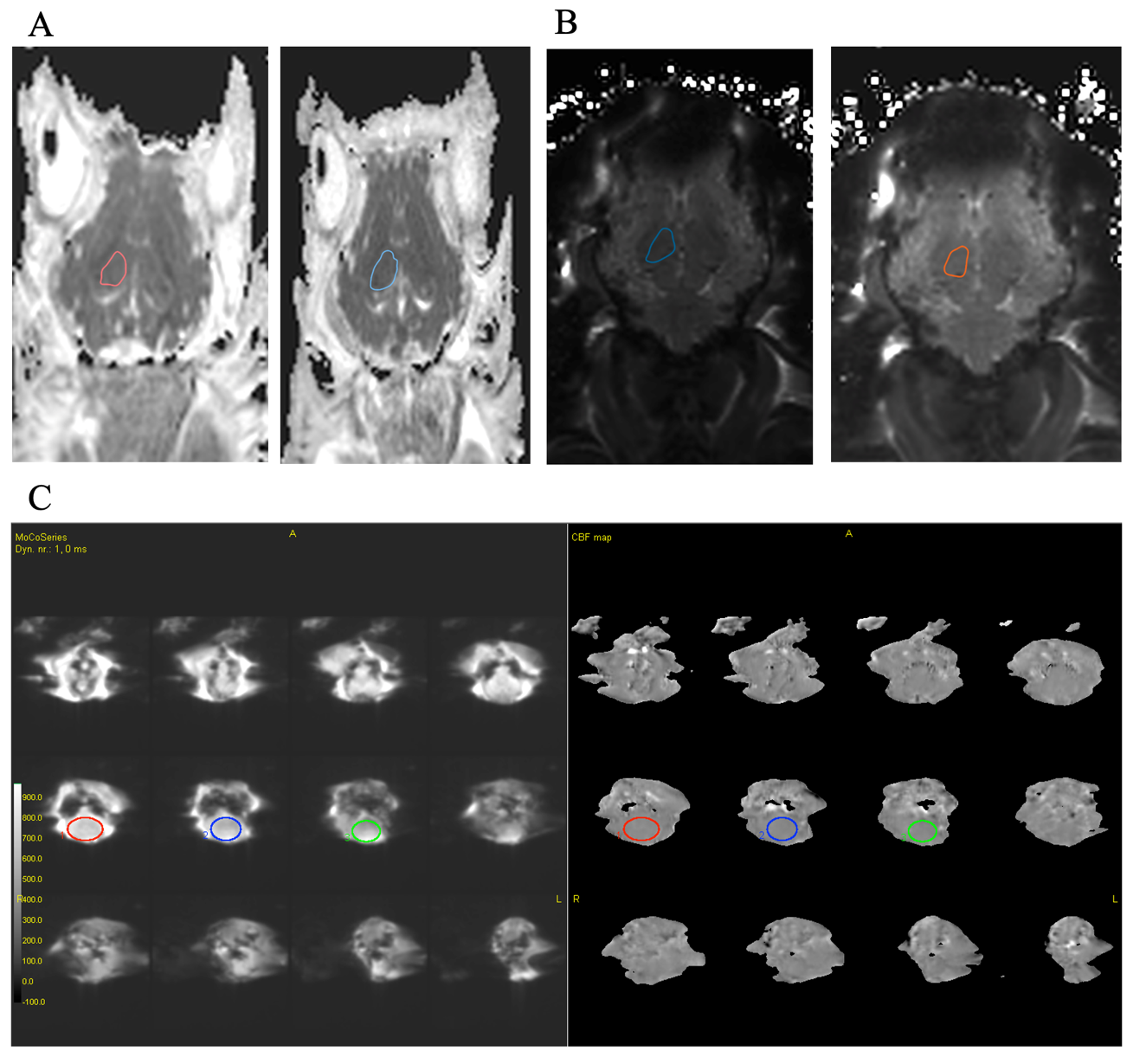


A) ADC maps, B) T2*-maps, C) ASL sequences

ADC, apparent diffusion imaging; ASL, arterial spin labeling.

**Supplementary 4. Anesthesia and inotropies in newborn piglets following lipopolysaccharide-sensitized hypoxic-ischemia.** Values are compared between piglets treated without (NT) and with therapeutic hypothermia (TH). Differences were analyzed by unpaired t-test and presented with mean values and standard deviations. Inotropies are also presented with number of piglets receiving. Use of cortisol was analyzed by Fisher’s exact test and presented solely as number of piglets receiving.

|  | NT group | TH group | *p*-value |
| --- | --- | --- | --- |
| Propofol (mg/kg/hour) | 2.5 (0.8) | 2.4 (1.0) | 0.94 |
| Fentanyl (μg/kg/hour) | 5.4 (1.6) | 5.4 (2.0) | 0.99 |
| Noradrenaline (total mg/kg) | 12/16 (0.77 (0.6)) | 14/16 (1.10 (0.4)) | 0.14 |
| Adrenaline (total mg/kg) | 4/16 (0.10 (0.05)) | 5/16 (0.13 (0.04) | 0.31 |
| Dobutamine (total mg/kg) | 4/16 (1.6 (1.4)) | 3/16 (2.1 (1.3)) | 0.61 |
| Methylprednisolone (n) | 3/16 (19 %) | 1/16 (6 %) | 0.60 |

**Supplementary 5. Magnetic resonance spectroscopy used to assess the NAA/Cr ratio in newborn piglets following lipopolysaccharide-sensitized hypoxia-ischemia.** Values are compared between piglets treated without (NT) and with therapeutic hypothermia (TH). Data were analyzed by unpaired t-test and presented with mean values and standard deviation. No statistically significant difference was found between groups.

**Supplementary 6. Magnetic resonance spectroscopy used to assess the NAA/Cho ratio in newborn piglets following lipopolysaccharide-sensitized hypoxia-ischemia.** Values are compared between piglets treated without (NT) and with therapeutic hypothermia (TH). Data were analyzed by unpaired t-test and presented with mean values and standard deviation. No statistically significant difference was found between groups.

**Supplementary 7. Amplitude-integrated encephalography score in newborn piglets following lipopolysaccharide-sensitized hypoxia-ischemia.** Values are compared between piglets treated without (NT) and with therapeutic hypothermia (TH). Data were analyzed by unpaired t-test and presented with mean values and standard deviation. No statistically significant difference was found between groups.

**Supplementary 8. Examples of immunohistochemical stainings in the thalamus for glial fibrillary acidic protein (GFAP), ionized calcium-binding adaptor molecule 1 (IBA1), and cleaved caspase-3 (CC3) in newborn piglets following lipopolysaccharide-sensitized hypoxia-ischemia.**

|  | **Normothermia** | **Hypothermia** |
| --- | --- | --- |
| **GFAP** | 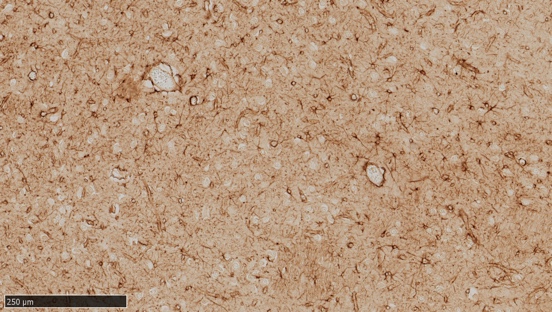 | 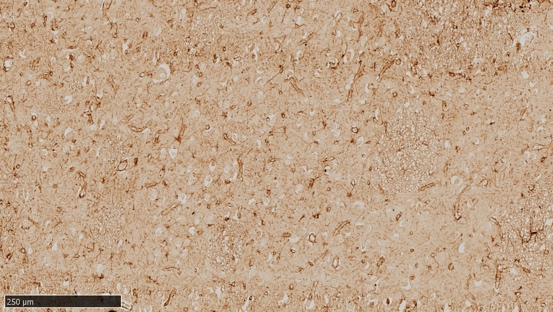 |
| **IBA1** | 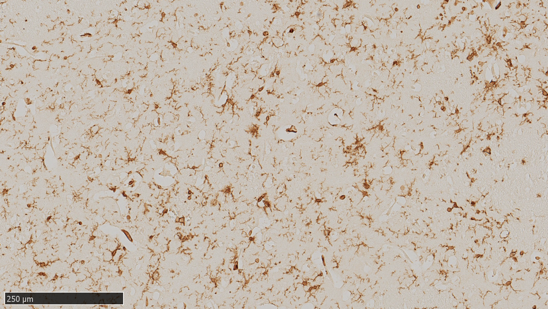 | 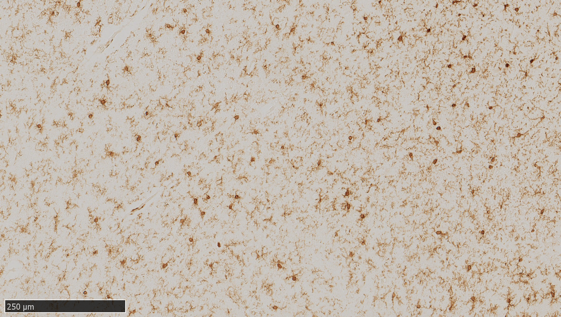 |
| **CC3** | 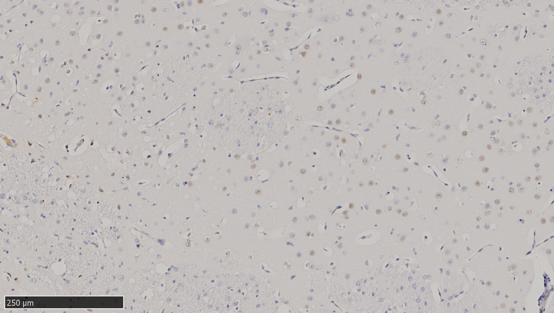 | 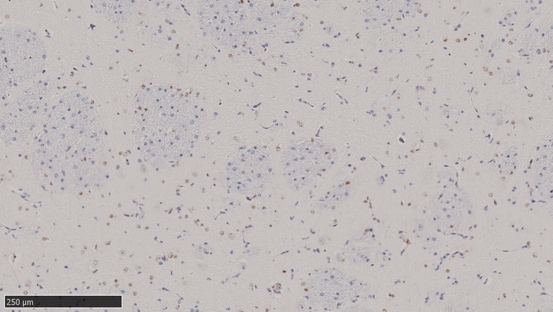 |

**Supplementary 9. Linear regression analyses with and without adjustment for blood glucose before scanning.** Results are presented with coefficients of therapeutic hypothermia (TH) and 95% confidence intervals (CI). Glial Fibrillary acidic protein (GFAP) and ionizing adaptor protein 1 (IBA1) are presented with stain density, while cleaved-caspase 3 is presented with number of positive cells.

Thalamus

*Log(Lac/NAA) ratio*

|  | Coefficient of TH | 95% CI |
| --- | --- | --- |
| Crude | 0.18 | -1.93 to 2.30 |
| Adjusted | -0.77 | -3.01 to 1.48 |

*GFAP*

|  | Coefficient of TH | 95% CI |
| --- | --- | --- |
| Crude | 0.6 | -11.5 to 12.7 |
| Adjusted | -3.7 | -17.8 to 10.4 |

*IBA1*

|  | Coefficient of TH | 95% CI |
| --- | --- | --- |
| Crude | 28 | -69 to 126 |
| Adjusted | 16 | -104 to 135 |

*CC3*

|  | Coefficient of TH | 95% CI |
| --- | --- | --- |
| Crude | -0.7 | -2.0 to 0.6 |
| Adjusted | -0.7 | -2.3 to 0.9 |

White matter

*Log(Lac/NAA) ratio*

|  | Coefficient of TH | 95% CI |
| --- | --- | --- |
| Crude | 0.60 | -1.78 to 2.98 |
| Adjusted | -0.41 | -2.98 to 2.16 |

*GFAP*

|  | Coefficient of TH | 95% CI |
| --- | --- | --- |
| Crude | 5.5 | -4.4 to 15.3 |
| Adjusted | 7.9 | -3.9 to 19.7 |

*IBA1*

|  | Coefficient of TH | 95% CI |
| --- | --- | --- |
| Crude | -45 | -197 to 107 |
| Adjusted | -109 | -278 to 61 |

*CC3*

|  | Coefficient of TH | 95% CI |
| --- | --- | --- |
| Crude | -0.1 | -0.6 to 0.5 |
| Adjusted | 0.1 | -0.6 to 0.7 |

Cortex

*Log(Lac/NAA) ratio (frontal)*

|  | Coefficient of TH | 95% CI |
| --- | --- | --- |
| Crude | 0.24 | -2.66 to 3.14 |
| Adjusted | -2.06 | -5.56 to 1.44 |

*Log(Lac/NAA) (occipital)*

|  | Coefficient of TH | 95% CI |
| --- | --- | --- |
| Crude | 0.67 | -1.95 to 3.30 |
| Adjusted | -1.26 | -4.15 to 1.62 |

*GFAP (parietal)*

|  | Coefficient of TH | 95% CI |
| --- | --- | --- |
| Crude | -5.2 | -19.1 to 8.6 |
| Adjusted | -8.9 | -25.4 to 7.7 |

*IBA1 (parietal)*

|  | Coefficient of TH | 95% CI |
| --- | --- | --- |
| Crude | -156 | -462 to 151 |
| Adjusted | -259 | -619 to 100 |

*CC3 (parietal)*

|  | Coefficient of TH | 95% CI |
| --- | --- | --- |
| Crude | -0.7 | -2.0 to 0.6 |
| Adjusted | -0.7 | -2.4 to 1.0 |
